# Supplementary figures and images for: 3D in situ imaging of the female reproductive tract reveals molecular signatures of fertilizing spermatozoa in mice
Source: eLife. 2020 Oct 20;9:e62043. doi: 10.7554/eLife.62043 (PMC7707823; doi:10.7554/eLife.62043)

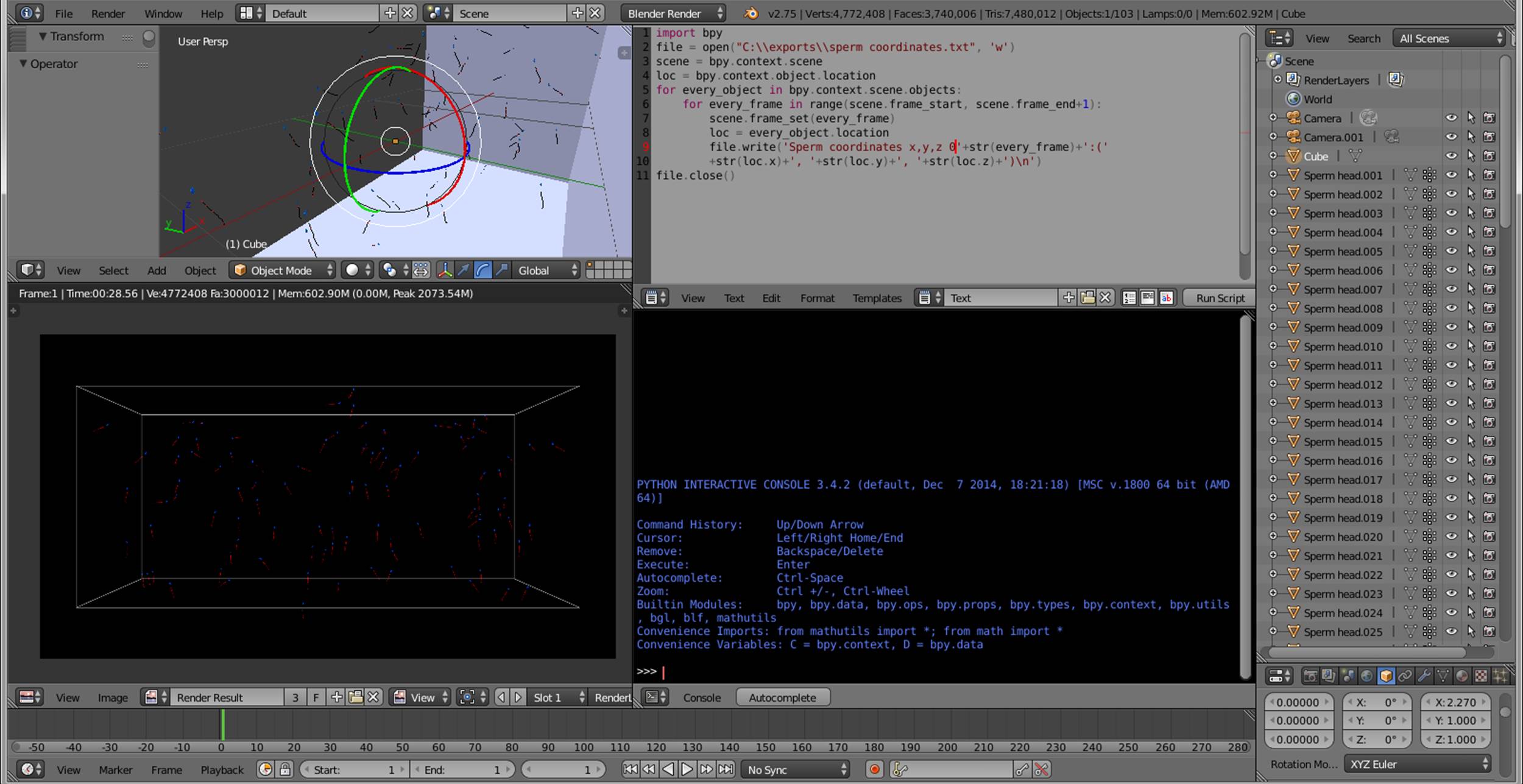

Supplement: Figure 5—source data 1. — Custom scripts and 3D objects for developing training environments, ANN input feeding and training, and constructing numerical objects coordinates arrays for supervised learning, and ANN performance evaluation. [file elife-62043-fig5-data1.zip › Sperm coordinates xyz export matrix generation for ANN supervised/PrtSc for exporting coordinates xyz of sperm in 3D space in Blender.jpg]

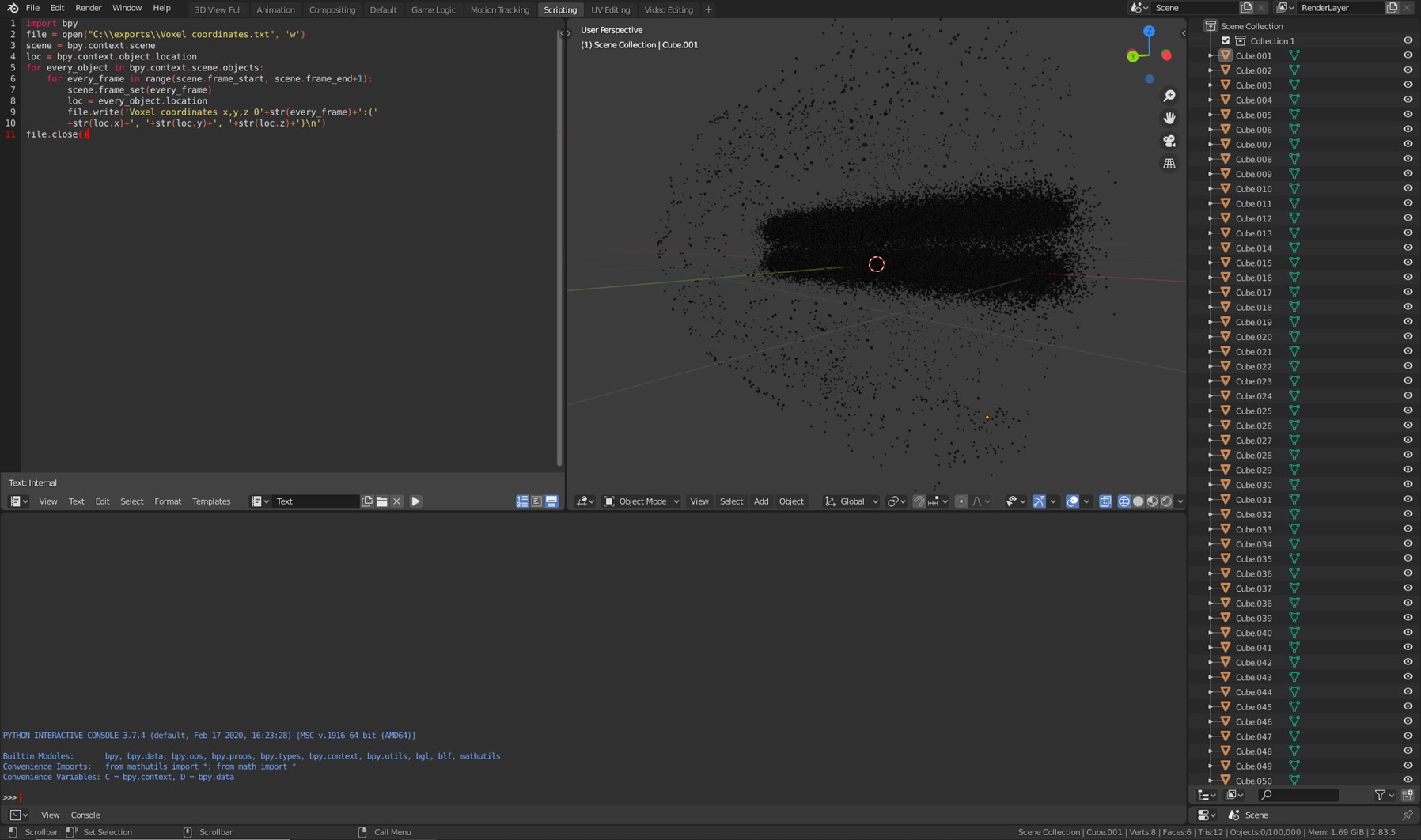

Supplement: Figure 6—source data 2. — Custom scripts for analyzing CatSper quadrilateral structure. [file elife-62043-fig6-data2.zip › PrtSc CatSper quadrilateral structure high poly voxel denstity n=100000 for mask n=80 application.jpg]

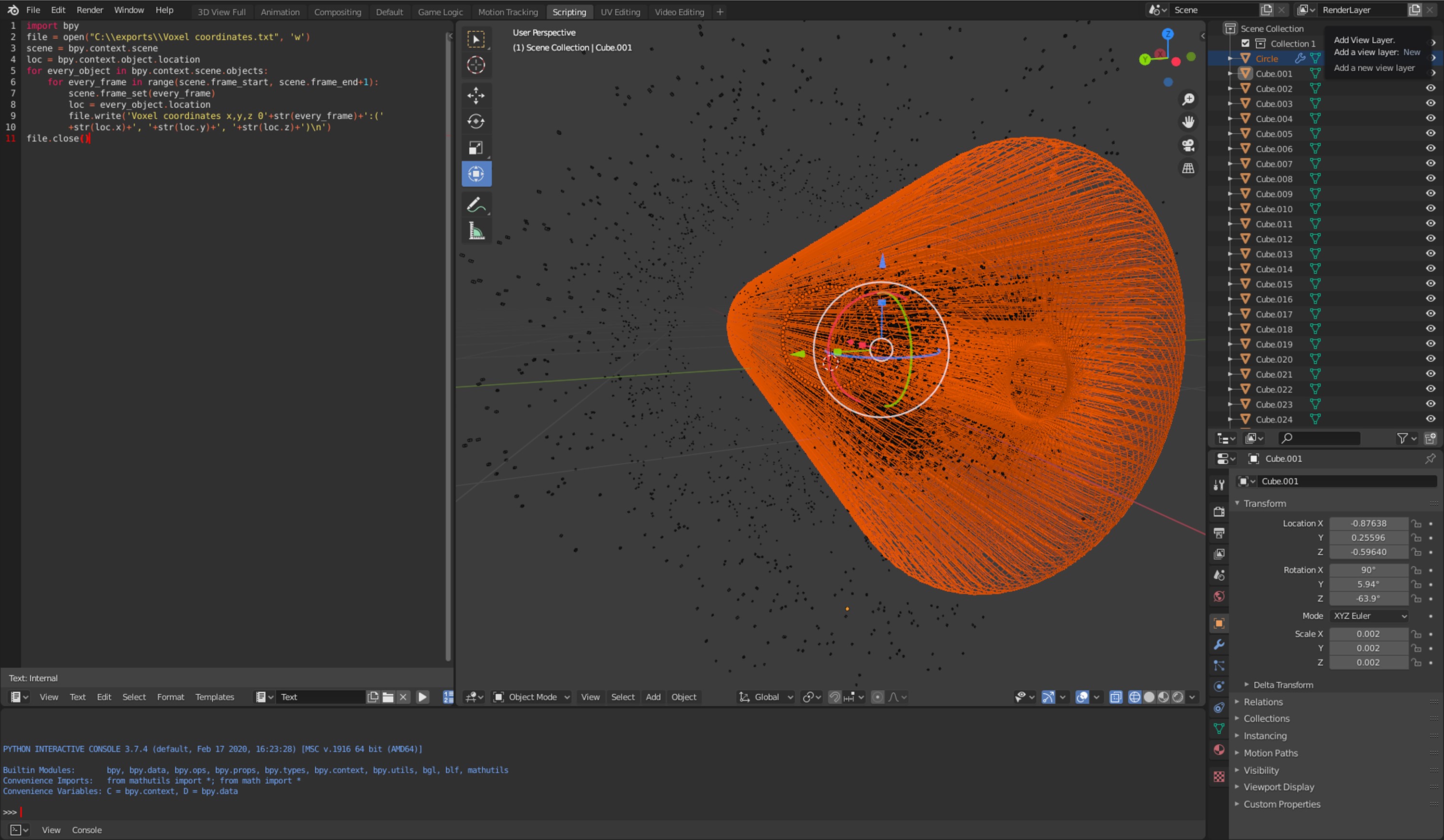

Supplement: Figure 6—source data 2. — Custom scripts for analyzing CatSper quadrilateral structure. [file elife-62043-fig6-data2.zip › PrtSc manual overlaying of the 3D masko n=80 over the imported CatSper domains topology n=100000.jpg]
