# Supplementary material for: 3D in situ imaging of the female reproductive tract reveals molecular signatures of fertilizing spermatozoa in mice
Source: eLife. 2020 Oct 20;9:e62043. doi: 10.7554/eLife.62043 (PMC7707823; doi:10.7554/eLife.62043)
Supplement: Transparent reporting form [file elife-62043-transrepform.docx]

***eLife’s* transparent reporting form**

We encourage authors to provide detailed information *within their submission* to facilitate the interpretation and replication of experiments. Authors can upload supporting documentation to indicate the use of appropriate reporting guidelines for health-related research (see [EQUATOR Network](http://www.equator-network.org/%20)), life science research (see the [BioSharing Information Resource](https://biosharing.org/)), or the [ARRIVE guidelines](http://www.plosbiology.org/article/info:doi/10.1371/journal.pbio.1000412) for reporting work involving animal research. Where applicable, authors should refer to any relevant reporting standards documents in this form.

If you have any questions, please consult our Journal Policies and/or contact us: [editorial@elifesciences.org](mailto:editorial@elifesciences.org).

**Sample-size estimation**

- You should state whether an appropriate sample size was computed when the study was being designed
- You should state the statistical method of sample size computation and any required assumptions
- If no explicit power analysis was used, you should describe how you decided what sample (replicate) size (number) to use

Please outline where this information can be found within the submission (e.g., sections or figure legends), or explain why this information doesn’t apply to your submission:

Information regarding sample sizes and statistical tests are given in each figure and/or figure legend whenever applicable. (1) Confocal imaging of *in vitro* sperm samples (that do not show details within single cells) contain multiple cells. N represents number of sperm cells used for classification and/or quantification (Figure 2C, 2F, Figure 4B). (2) Some representative confocal images CatSper1 in small number of sperm cells (that show the details within single cell level, Figure 2D) from microdissection were shown with no explicit power of analysis. This is justified because we tested the same hypothesis at multiple levels (*in vitro*, *in vivo/in situ*) and finally, the ANN analysis in the later part of the manuscript itself (Figure 5 and 6) from in situ imaging provides objective, robust, and automatic quantitation to support the conclusions from the former part of the study.

**Replicates**

- You should report how often each experiment was performed
- You should include a definition of biological versus technical replication
- The data obtained should be provided and sufficient information should be provided to indicate the number of independent biological and/or technical replicates
- If you encountered any outliers, you should describe how these were handled
- Criteria for exclusion/inclusion of data should be clearly stated
- High-throughput sequence data should be uploaded before submission, with a private link for reviewers provided (these are available from both GEO and ArrayExpress)

Please outline where this information can be found within the submission (e.g., sections or figure legends), or explain why this information doesn’t apply to your submission:

All the biochemistry and imaging experiments were repeated at least three times with the samples prepared independently from 3-4 different animals. The number of all experiments are given in each figure legend when representative images are shown.

For ANN analysis, all the individual data points (N) used for analysis are given and displayed to show the raw data distribution.

**Statistical reporting**

- Statistical analysis methods should be described and justified
- Raw data should be presented in figures whenever informative to do so (typically when N per group is less than 10)
- For each experiment, you should identify the statistical tests used, exact values of N, definitions of center, methods of multiple test correction, and dispersion and precision measures (e.g., mean, median, SD, SEM, confidence intervals; and, for the major substantive results, a measure of effect size (e.g., Pearson's r, Cohen's d)
- Report exact p-values wherever possible alongside the summary statistics and 95% confidence intervals. These should be reported for all key questions and not only when the p-value is less than 0.05.

General information on statistical analyses is given at the end of Materials and Methods part. Detailed tests and data representation for quantification results including *P*-values are described in each figure legend. Actual numerical data for figures that are represented in graphs are all provided as source data.

Please outline where this information can be found within the submission (e.g., sections or figure legends), or explain why this information doesn’t apply to your submission:

(For large datasets, or papers with a very large number of statistical tests, you may upload a single table file with tests, Ns, etc., with reference to sections in the manuscript.)

**Group allocation**

- Indicate how samples were allocated into experimental groups (in the case of clinical studies, please specify allocation to treatment method); if randomization was used, please also state if restricted randomization was applied
- Indicate if masking was used during group allocation, data collection and/or data analysis

N/A

Please outline where this information can be found within the submission (e.g., sections or figure legends), or explain why this information doesn’t apply to your submission:

**Additional data files (“source data”)**

- We encourage you to upload relevant additional data files, such as numerical data that are represented as a graph in a figure, or as a summary table
- Where provided, these should be in the most useful format, and they can be uploaded as “Source data” files linked to a main figure or table
- Include model definition files including the full list of parameters used
- Include code used for data analysis (e.g., R, MatLab)
- Avoid stating that data files are “available upon request”

Please indicate the figures or tables for which source data files have been provided:

We have provided source data files containing numerical data for graphs with inferential statistics (Figure 1G, Figure 4B, Figure 6H). We have provided a source data file containing numerical data and statistical analyses for a graph that is represented without statistics due to space constraint (Figure 2F). The ANN procedure is performed by MatLab9.3 (R2017b) software ANN toolbox as described in Materials and Method. The workflow is described in Figure 5-figure supplement 2. We have included training 3D environments (.obj/.fbx files) and the custom scripts (text/Notepad++ files) for changing of the object abundancies and object transformations for Phython as source data for Figure 5 and Figure 6.
